# Myocyte Enhancer Factor 2A (MEF2A) Defines Oxytocin-Induced Morphological Effects and Regulates Mitochondrial Function in Neurons

**DOI:** 10.3390/ijms21062200

**Published:** 2020-03-23

**Authors:** Magdalena Meyer, Kerstin Kuffner, Julia Winter, Inga D. Neumann, Christian H. Wetzel, Benjamin Jurek

**Affiliations:** 1 Department of Behavioral and Molecular Neurobiology, University of Regensburg, 93040 Regensburg, Germany; Magdalena.Meyer@ur.de (M.M.); julia.winter@ur.de (J.W.); inga.neumann@ur.de (I.D.N.); 2 Department of Psychiatry and Psychotherapy, University of Regensburg, 93040 Regensburg, Germany; kerstin.kuffner@ukr.de (K.K.); Christian.Wetzel@ukr.de (C.H.W.)

**Keywords:** oxytocin, morphology, neurite outgrowth, neurite retraction, autism, MEF2A, CRISPR-Cas, hyperconnectivity

## Abstract

The neuropeptide oxytocin (OT) is a well-described modulator of socio-emotional traits, such as anxiety, stress, social behavior, and pair bonding. However, when dysregulated, it is associated with adverse psychiatric traits, such as various aspects of autism spectrum disorder (ASD). In this study, we identify the transcription factor myocyte enhancer factor 2A (MEF2A) as the common link between OT and cellular changes symptomatic for ASD, encompassing neuronal morphology, connectivity, and mitochondrial function. We provide evidence for MEF2A as the decisive factor defining the cellular response to OT: while OT induces neurite retraction in MEF2A expressing neurons, OT causes neurite outgrowth in absence of MEF2A. A CRISPR-Cas-mediated knockout of MEF2A and retransfection of an active version or permanently inactive mutant, respectively, validated our findings. We also identified the phosphatase calcineurin as the main upstream regulator of OT-induced MEF2A signaling. Further, MEF2A signaling dampens mitochondrial functioning in neurons, as MEF2A knockout cells show increased maximal cellular respiration, spare respiratory capacity, and total cellular ATP. In summary, we reveal a central role for OT-induced MEF2A activity as major regulator of cellular morphology as well as neuronal connectivity and mitochondrial functioning, with broad implications for a potential treatment of disorders based on morphological alterations or mitochondrial dysfunction.

## 1. Introduction

Oxytocin (OT) is a neuropeptide profoundly implicated in the regulation of various socio-emotional behaviors and physiological responses [1,2]. Dysregulated plasma or salivary levels of OT have been associated with symptoms of autism spectrum disorder (ASD) [3,4], depression [5], and anxiety [6], and its intranasal application has been described to relieve some of those symptoms [7]. In particular, the pro-social effects of OT, as described by multiple groups [8,9,10,11,12], promise to relieve core deficits in social behavior in ASD patients. As a multifactorial disorder, ASD has been associated with neuronal, morphological and mitochondrial dysfunctions [13,14,15,16,17]. One common factor between ASD, its underlying disturbed cellular functions and the oxytocinergic system, is the transcription factor myocyte enhancer factor (MEF) 2. In this study, we provide evidence for the role of MEF2 as the central node in the regulation of those conditions.

One major parameter in ASD patients is an alteration of the neuronal connectivity. Autistic patients show hyperconnectivity between amygdala and hippocampus, which is negatively correlated with peripheral OT levels, and can even be further decreased by intranasal application of OT [4]. Although neuronal cell types differ between brain regions, i.e., hypothalamus and amygdala, the underlying molecular mechanism that connects OT receptor-coupled signaling with neuronal morphology seems to be identical in various cell types ([1] and references therein). In particular, transcriptomic responses to OT are similar in hypothalamic as well as amygdalar neurons [18]. Furthermore, neurons generated from iPSCs of ASD patients have more complex and longer neurites, causing increased synapse number and a state of hyperconnectivity [19,20]. MEF2 is one factor that is responsible for the regulation of excitatory synapse number [21,22,23], and we have recently provided evidence for its activation by OT receptor-coupled signaling pathways [24]. The activation of Gq protein-coupled OT receptors leads to an increase in intracellular calcium from either intracellular stores [25], through cation channels such as TrpV2/4 [26,27] or L-type voltage-gated calcium channels [28]. Upon OT-induced calcium influx, the phosphatase calcineurin (CaN, or protein phosphatase 2B; PP2B) is activated by binding calmodulin [29]. On the one hand, active CaN acts as a direct phosphatase of cytoskeletal proteins and influences the stability and interaction of microtubule and actin filaments [30,31,32]. On the other hand, it dephosphorylates myocyte enhancer factor 2A (MEF2A) at position S408 [21,22], leading to a genomic response that consequentially impacts cellular morphology indirectly via transcriptional control of regulators of the cytoskeleton. Such regulators have recently been identified and linked to OT signaling by Bakos and colleagues, who found increased expression of the actin-binding proteins drebrin, the intermediate filament vimentin, or the scaffolding protein SHANK3, under the influence of OT. This caused neurite outgrowth in human SH-SY5Y neuroblastoma or U-87MG glioblastoma cells [28,33,34]. As a consequence of neurite outgrowth, altered connectivity is observable due to increased synapse formation, which is under the OT-induced control of synaptic adhesion molecules such as neuroligin 3 or neurexin 2 [35]. Since OT is produced in the hypothalamus and we have found profound anxiolytic and anti-stress effects of OT infused into the hypothalamic paraventricular nucleus [18,36,37,38], we aimed to determine the intracellular effects of OT in a hypothalamic cell line, as opposed to neuroblastoma or glioblastoma cell types. As a cellular model of OTergic effects, we have characterized the rat hypothalamic neuronal cell line H32, which expresses MEF2A endogenously, and retract their neurites upon OT stimulation, caused by MEF2A activation [24]. Neurite retraction was blocked by siRNA-mediated knockdown of MEF2A, or pharmacological blockade of the OT receptor-induced activation of the mitogen-activated protein kinase (MAPK) pathway by U0126. The observed neurite retraction was seemingly in contrast to the above-mentioned studies showing OT-induced neurite outgrowth in SH-SY5Y or U-87MG cells, but these are not hypothalamic neurons and might therefore react differently. In support of this hypothesis, we characterized the transcriptome of SH-SY5Y cells and found absent MEF2A expression. This phenotypical contrast led us to create a MEF2A knockout cell line, derived from our MEF2A-positive H32 cells (H32ΔMEF2A), using CRISPR-Cas9. In addition, we characterized another hypothalamic cell line (mHypoE-N11), which does not express endogenous MEF2A, and used those cells to overexpress MEF2A by plasmid transfection. By site-directed mutagenesis, we substituted the serine at position 408 to aspartate, creating a permanent phospho-mimetic MEF2A mutant. This mutant, transfected into H32ΔMEF2A cells, served as control for phospho-site-specific effects of MEF2A, since MEF2A can be phosphorylated at three different residues, namely S408, Thr312, and Thr319, with opposing outcomes for gene transcription [39]. Those three hypothalamic cell lines serve as internal controls in our experimental setup and aid in determining the morphological outcome of OT stimulation in hypothalamic cells in the presence or absence of MEF2A. Cytoskeletal rearrangements are energy-consuming events, requiring proper functioning of mitochondria. The organelles produce adenosine triphosphate (ATP) and provide energy for several cellular processes in neurons including action potential generation, but also morphological changes such as neurite growth. Many neurodegenerative diseases with a loss of neuronal function and morphology show accompanying mitochondrial malfunctions [40]. Intriguingly, MEF2 regulates transcription of the mitochondrial NADH dehydrogenase 6 gene, which is essential for the function of the oxidative phosphorylation system [41,42], which ultimately regulates the production of ATP. In line with that, a significant proportion of ASD patients suffer from abnormal ATP production [43,44], suggesting a central role for the OT-regulated transcription factor MEF2 in energy balance, structural plasticity, and ASD (also see [22,24,45,46,47]). To test this hypothesis, we manipulated MEF2A activity in hypothalamic neurons and monitored morphological alterations and mitochondrial functionality.

## 2. Results

In order to study the impact of MEF2A in OT-induced effects on cellular morphology, we used cell lines that lacked, endogenously expressed, or were transfected with MEF2A. The mother cell line as well as all genetically altered daughter lines express the OT receptor, as validated by RT-qPCR.

The hypothalamic rat cell line H32 was subjected to CRISPR-Cas-mediated knockout of MEF2A (H32ΔMEF2A), and retransfected with either wild-type rat MEF2A (H32ΔMEF2A^MEF2A^) or the mutant version H32ΔMEF2A^MEF2A[S408D]^, in which site-directed mutagenesis at S408D mimicked a permanently phosphorylated, and therefore transcriptionally inactive transcription factor.

The mouse hypothalamic cell line mHypoE-N11 lacked MEF2A expression with levels below the detection limit of a qPCR. Transfection of those cells with a plasmid encoding for mouse MEF2A resulted in exogenous overexpression of MEF2A, as verified with immunofluorescence.

The cell lines created and their MEF2A expression profile are summarized in Table 1.

**Figure 1:**
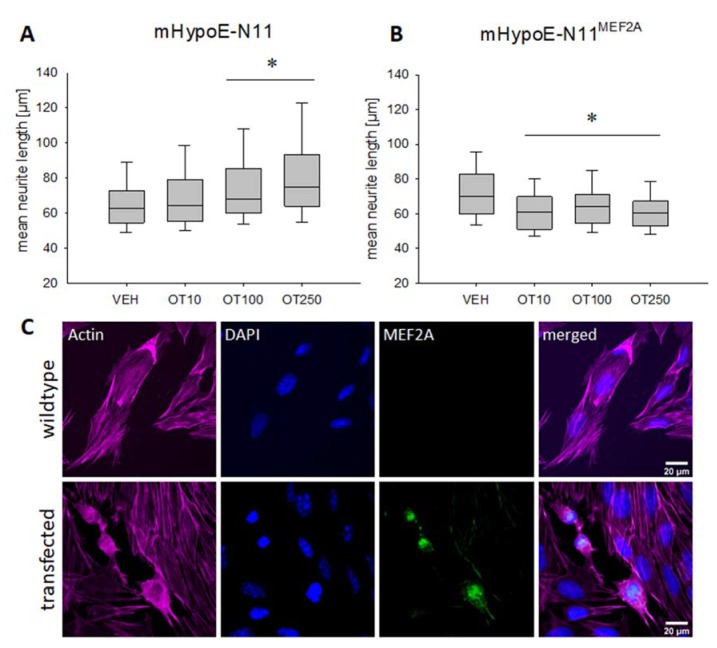
Effects of oxytocin (OT) on neuronal morphology in the mouse hypothalamic cell line mHypoE-N11. (**A**) Median neurite length increases significantly in wild-type mHypoE-N11 cells treated overnight with 100 or 250 nM OT. *n* = 100 cells per treatment; * *p* < 0.012 vs. VEH. (**B**) Treatment with 10, 100 and 250 nM of OT induces a significant neurite retraction in MEF2A overexpressing mHypoE-N11 cells. *n* = 100 cells per treatment; * *p* < 0.003 vs. VEH. (**C**) Representative images of immunofluorescence labelling for MEF2A. Images were sequentially recorded for F-actin (Phalloidin, red, first column), chromatin (DAPI, blue, second column), and MEF2A (green, third column). Merged images are shown for each row in the fourth column. Labels left to the columns indicate the respective cell type (wild-type vs. transfected).

Treatment of mouse hypothalamic MEF2A-deficient mHypoE-N11 cells (see Table 1), with increasing concentrations (10, 100 and 250 nM) of OT overnight, led to a dose-dependent increase in neurite length, which reached significance in the 100 and 250 nM treatment group, but not in the 10 nM group (Figure 1A). Statistical analyses for all figures are summarized in the Supplementary Materials, including details on p values and effect sizes.

Overexpressing MEF2A in mHypoE-N11 cells by plasmid transfection with subsequent OT stimulation 48 h later revealed a significant OT-induced retraction of neurites after 12 h in all doses tested (Figure 1B). Successful overexpression of MEF2A was controlled by immunostaining with an MEF2A-specific antibody (Figure 1C). As expected, we found mainly nuclear location of MEF2A, with partial cytosolic location, representing a normal localization of the transcription factor [39].

Neither of the effects on neurite length was caused by altered cell viability. We found no decrease in cellular viability in H32 or H32ΔMEF2A cells, and no increased cell viability in mHypoE-N11 cells under the influence of OT (Figure A2). This suggests that the OT-induced morphological effects are primarily alterations of the cytoskeleton, and not mere side effects of an apoptotic or otherwise constricted cell. The OT receptor specificity of the morphological effect has been addressed by the use of a specific OT receptor antagonist (des-Gly-NH2d(CH2)5[Tyr(Me)2Thr4]-OVT) and agonist (Thr4 Gly7-OT, TGOT) in a previous publication [24].

Having established the ‘gain-of-function-MEF2A’ model with the mHypoE-N11 cells, we determine whether a ‘loss-of-MEF2A-function’ model would reverse the OTergic effect on cellular morphology. To obtain a permanent knockout of MEF2A, we made use of the CRISPR-Cas9 technology to create a monoclonal knockout cell line derived from a single edited H32 clone. The efficiency of the knockout was validated by Western blotting.

**Figure 2:**
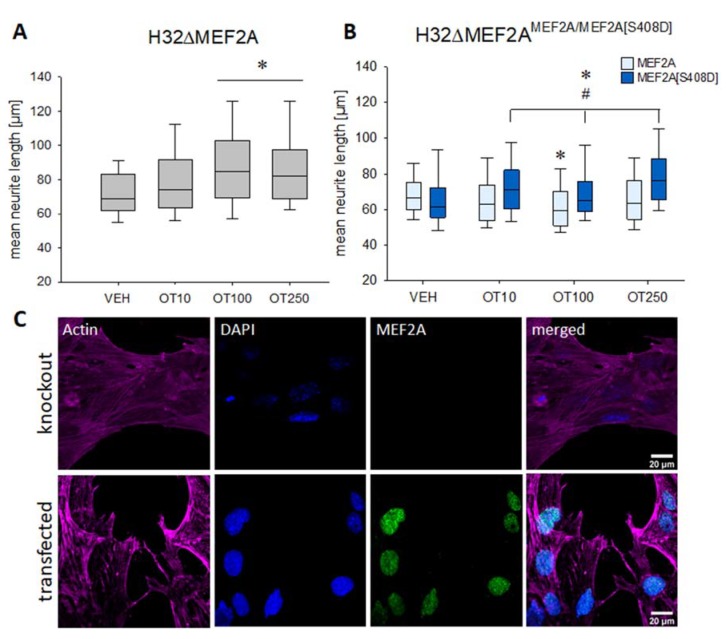
Effects of OT on neuronal morphology in the rat hypothalamic cell line H32. (**A**) Incubation of H32ΔMEF2A cells with 100 or 250 nM OT leads to a significant neurite elongation. *n* = 100 cells per treatment; * *p* < 0.001 vs. VEH. (**B**) H32ΔMEF2A cells were retransfected with wild-type MEF2A and a mutant version MEF2A[S408D], which mimics a permanently phosphorylated and therefore inactive MEF2A. The wild-type MEF2A generated OT-induced neurite retraction, whilst the mutant MEF2A lacked the ability to induce neurite retraction. Instead, the mutant response to OT resembled the MEF2A knockout cells. *n* = 100 cells per treatment; two-way ANOVA; * *p* < 0.031 treatments vs. VEH within the same group; # *p* < 0.002 groups (wild-type vs. mutant) within the same treatment. (**C**) Representative images of immunofluorescence labelling for MEF2A. Images were sequentially recorded for F-actin (Phalloidin, red, first column), chromatin (DAPI, blue, second column), and MEF2A (green, third column). Merged images are shown for each row in the fourth column. Labels left to the columns indicate the respective cell type (knockout vs. transfected).

In contrast to OT-induced neurite retraction in H32 wild-type cells, we found neurite elongation after stimulating H32ΔMEF2A cells overnight with 100 and 250 nM OT (Figure 2A). Retransfection of those knockout cells with an intact wild-type MEF2A reversed the effect and initiated OT-induced neurite retraction (Figure 2B, light blue columns). However, when a phospho-mimetic, permanently inactive MEF2A [S408D] mutant was retransfected, neurite elongation was observed (Figure 2B, dark blue columns). This cellular response was comparable to the H32ΔMEF2A cells (Figure 2A), revealing the S408 residue as the main phosphorylation site that orchestrates the OT effect on cellular morphology.

We have shown in a previous publication (Meyer et al., 2018) that incubation of H32 cells with 100 nM OT overnight led to a dephosphorylation and, therefore, activation, of MEF2A at S408, compared to VEH treated cells. This effect is indirectly mediated via the MAPK pathway, as it is reversed to basal by the MEK1/2 inhibitor U0126. This treatment does not alter the level of total MEF2A or MEK1/2 protein, as previously published by our group [18,24,26,37,38].

**Figure 3:**
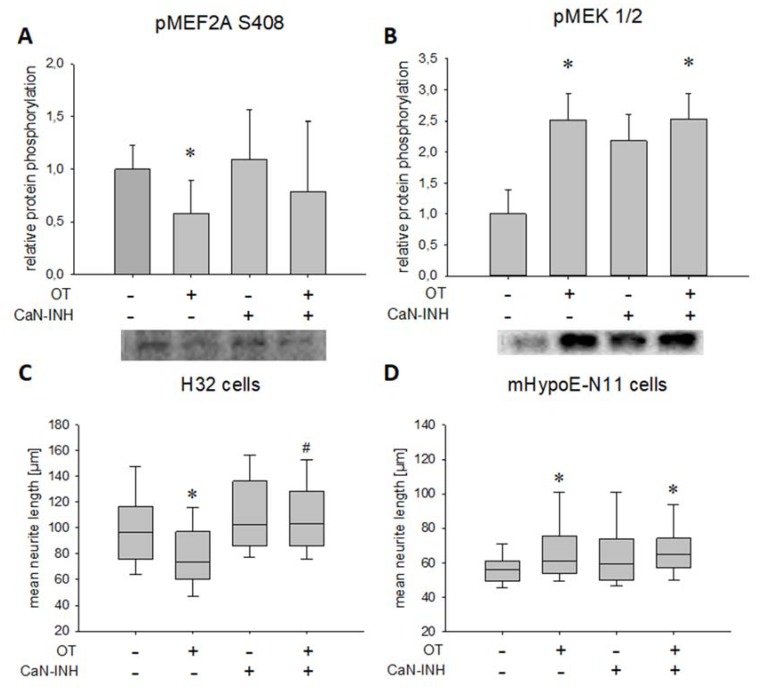
Effect of OT stimulation and calcineurin inhibition on phosphorylation of S408 of MEF2A and MEK1/2 as well as on the median neurite length in H32 cells. (**A**) Relative MEF2A S408 phosphorylation analyzed by Western blotting in whole cell lysate after treatment with VEH and OT, in the presence or absence of a CaN inhibitor (CaN-INH), with representative blot below. Overnight incubation of H32 wild-type cells with 100 nM OT led to a significant dephosphorylation of MEF2A at S408 compared to VEH treated cells (one-way ANOVA, *p* = 0.056; independent t-test VEH vs. OT, *p* = 0.006). This effect is dependent on CaN activity, as it is reversed to basal by a CaN-INH. The inhibitor without OT had no effect on MEF2A phosphorylation. *n* = 6 per treatment. (**B**) Relative phosphorylation of MEK1/2 analyzed by Western blotting in whole cell lysate after treatment with VEH and OT, in the presence or absence of a CaN inhibitor, with representative blot picture below. Treatment of the cells with 100 nM OT overnight led to a significant phosphorylation of MEK1/2 compared to VEH. Statistical analysis using one-way ANOVA revealed no significant effect of the inhibitor treated cells, but a significant phosphorylation of MEK1/2 when cells were treated with OT and the CaN-INH. *n* = 6 per treatment; * *p* < 0.05 vs. VEH. (**C**) In H32 cells, 100 nM of OT led to a significant retraction compared to VEH, while inhibition of CaN resulted in elongation of neurites overnight compared to VEH and OT. Combining the OT treatment with a CaN-INH increased the neurite length significantly. *n* = 100 per treatment; * *p* < 0.001 vs. VEH-treated cells; # *p* < 0.001 vs. OT-treated cells. (**D**) Incubation of mHypoE-N11 cells with 100 nM OT overnight led to an increased neurite length. Inhibition of CaN had no effect on the neuronal morphology, in line with the absent MEF2A expression in this cell line. *n* = 50 per treatment; * *p* < 0.05 vs. VEH-treated cells.

In the present study, OT stimulation led to decreased MEF2A S408 phosphorylation, whereas addition of CaN inhibitor reversed the phosphorylation back to basal (Figure 3A).

To test whether the observed CaN activation is related to the MAPK pathway, we assessed MEK1/2 phosphorylation after OT/CaN inhibitor treatment. Western blotting revealed MAPK pathway activation after OT treatment irrespective of the CaN inhibitor application, indicated by persistent MEK1/2 phosphorylation in the presence or absence of the CaN inhibitor. The inhibitor alone, despite a visual increase, had no significant effect on MEK1/2 phosphorylation (Figure 3B). As expected, those effects translated into morphological changes, i.e., OT treatment (as shown in Figure 2B and [24]) reduced neurite length from approximately 100 µm to approximately 75 µm, an effect that was blocked by the CaN inhibitor (Figure 3C). In line with this data, when CaN was blocked in mHypoE-N11 cells (which do not express MEF2A), OT retained its ability to increase neurite length (Figure 3D). This is in line with our hypothesis of the central role for the CaN-MEF2A pathway in OT-induced neurite retraction, but OT induces neurite outgrowth when MEF2A is absent.

**Figure 4:**
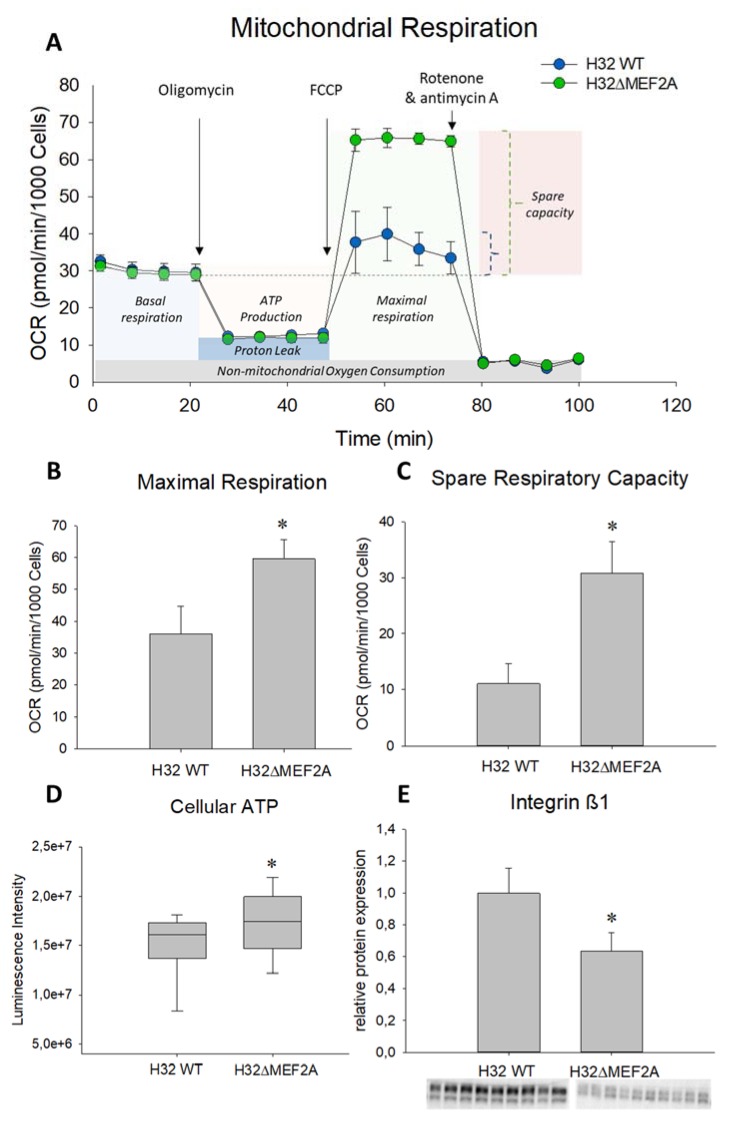
Effect of MEF2A knockout on mitochondrial function and neuronal connectivity. (**A**) The Agilent Seahorse XF Cell Mito Stress Test profile shows the oxygen consumption rate (OCR) in H32 wild-type (WT) and H32ΔMEF2A cells representing key parameters of mitochondrial function. (**B**) and (**C**) The OCR of both cell lines in the mitochondria stress test measured by Seahorse. H32ΔMEF2A cells show a significantly increased maximal respiration (* *p* = 0.004) and spare respiratory capacity (maximal vs. basal respiration, * *p* = 0.01) compared to H32 cells. *n* = 3 test repetitions with 4 measurement replicates per group. (**D**) Cellular ATP content under basal conditions is significantly higher in H32ΔMEF2A than H32 WT cells. *n* = 24 per group; * *p* = 0.038. (**E**) Relative protein expression of integrin ß1 is significantly downregulated in H32ΔMEF2A cells compared to H32 WT cells. n (H32 WT) = 9, n (H32ΔMEF2A) = 10; * *p* < 0.001.

For examination of mitochondrial function, we analyzed intact H32 wild type and H32ΔMEF2A cells with the Agilent Seahorse XF Cell Mito Stress Test Kit. After application of different stressors to the cells, the oxygen consumption rate was measured, which serves as an indicator for mitochondrial functioning (Figure 4A). H32ΔMEF2A cells showed a significantly higher maximal respiration (Figure 4B) as well as spare respiratory capacity (Figure 4C) compared to the MEF2A expressing H32 wild-type cells. This increased respiratory capacity in the H32ΔMEF2A cells suggests an inhibitory role for MEF2A in mitochondrial functioning. As a direct consequence of this altered mitochondrial performance, basal cellular ATP content was significantly elevated in the H32ΔMEF2A cells, when compared with the wild-type cells (Figure 4D). This additional energy supply might contribute to the cytoskeletal rearrangements that cause neurite elongation observed under OT treatment.

Neurite outgrowth serves to increase cell–cell contacts, which we hypothesized to simultaneously decrease cell–matrix contacts [49,50]. As a representative indicator for decreased cell–matrix contacts, we assessed integrin ß1 receptor expression [51], and found decreased protein expression in H32ΔMEF2A cells compared to H32 wild-type cells (Figure 4E).

## 3. Discussion

**Figure 5:**
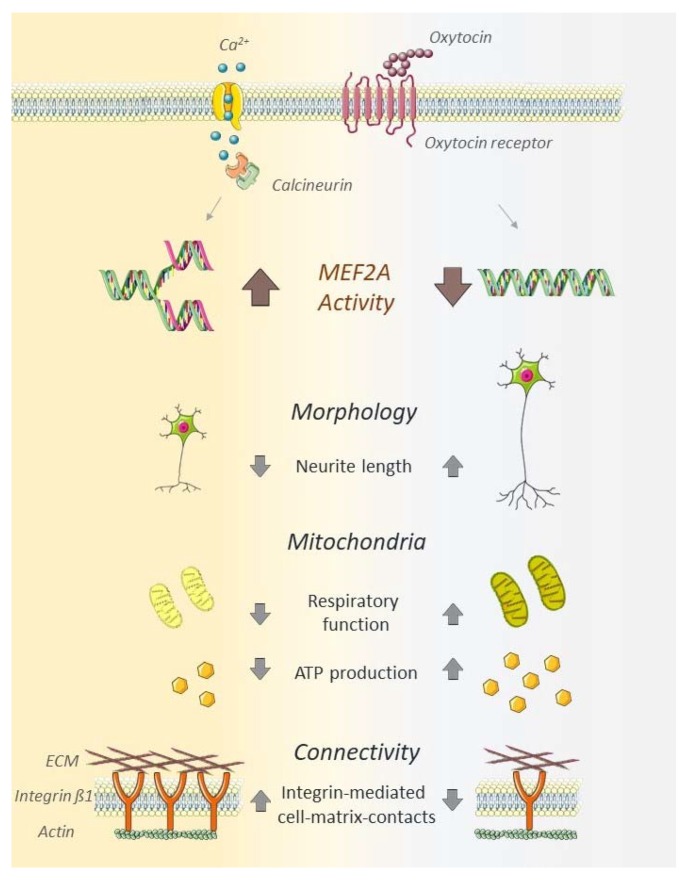
Schematic summary of MEF2A activity in OT-stimulated neurons. Neuronal MEF2A activity, induced by OT, reduces neurite length, decreases mitochondrial respiratory function, decreases ATP production, and increases integrin β1-mediated cell–matrix contacts. The absence or inhibition of MEF2A induces neurite outgrowth, increased respiratory function, ATP production, and decreased cell–matrix connectivity. The schematic art pieces used in this figure were provided by Servier Medical art (http://servier.com/Powerpoint-image-bank). Servier Medical Art by Servier is licensed under a Creative Commons Attribution 3.0 Unported License.

In recent publications, OT has been considered as a potential treatment for the alleviation of adverse psychiatric traits, e.g., ASD-related symptoms [7,52,53,54]. The effects range from improving social skills in autistic children [55,56] to increased reciprocal trust in adult healthy probands [53]. However, the underlying molecular mechanism of the OT’s alleviating properties on the symptoms of ASD are not fully understood. Considering the cellular changes apparent in ASD patients, we focused on the main parameters of cellular morphology, mitochondrial function and neuronal connectivity in this study, providing evidence for MEF2A as central regulator of those processes (Figure 5).

We have identified the transcription factor MEF2A as the defining parameter that shifts the OT-induced neuronal response from neurite outgrowth towards neurite retraction. Knockin or knockout of MEF2A produced dichotomic effects on neuronal morphology, but only in the presence of OT. When MEF2A is expressed, OT leads to transcriptional activation by dephosphorylation of the inhibitory site S408 of MEF2A, which promotes a switch from sumoylation to acetylation at Lys403 [22] and subsequent inhibition of dendritic morphogenesis [57]. In contrast, when we transfected MEF2A knockout cells with the transcriptionally inactive mutant (H32ΔMEF2A^MEF2A[S408D]^), sumoylation at Lys403 was retained and dendritic morphogenesis occurred. Interestingly, the knockout of MEF2A and its phospho-mimetic mutation did not just inhibit neurite retraction, but actively induced neurite outgrowth under the influence of OT. This implies alternative OTR-coupled pathways, which are silenced or overruled when MEF2A is expressed and active. One potential pathway is the MAPK pathway, which is activated by the OTR, but not affected by the MEF2A knockout or inhibition of CaN. The MAPK pathway has been associated with OT-induced anxiolysis, i.e., reduction of anxiety-like behavior in rats [18,26,36,37,38] and targets transcription factors such as CREB and its cofactor CRTC3 [18,58]. The MAPK pathway is certainly one of the main effectors of OTR signaling [1]; however, it might not be directly linked to MEF2A activation, mainly because the S408 residue requires dephosphorylation, not phosphorylation, for full transcriptional activation. Evidence from hippocampal neurons suggests that a calcium-dependent activation of MEF2A requires the CaN-dependent dephosphorylation of MEF2A [21,22]. Based on this data, we identified CaN as the central part of a signaling cascade coupled to the OTR and thereby influencing the morphological response. Inhibiting CaN diminished the OT-induced neurite retraction in the H32 cells, strongly suggesting CaN as the upstream phosphatase responsible for OT-induced MEF2A activation. Moreover, mHypoE-N11 cells that lack MEF2A as a target of CaN should not be affected by CaN inhibition. Indeed, blocking CaN activity did not interfere with OT-induced (MEF2A-independent) neurite elongation. Since the actions of CaN and the MAPK signaling pathway have been shown to be co-dependent [59,60,61], we tested MEK1/2 activity under OT/CaN inhibition. In line with our hypothesis, OT-induced MEK1/2 phosphorylation was not affected by CaN inhibition, implying a direct activation of CaN by OTR-mediated Ca^2+^ influx through either TrpV2 channels [26], or release from intracellular stores ([1] and references therein).

**Figure A1:**
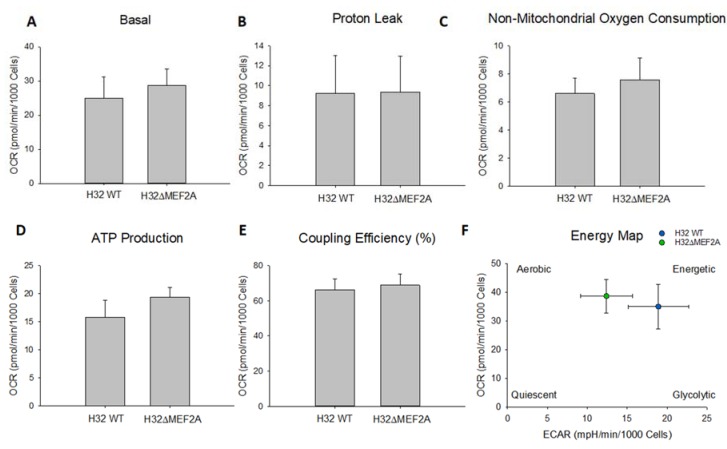
Mitochondrial function in H32 wild-type and H32ΔMEF2A cells. *n* = 3 per group.

**Figure A2:**
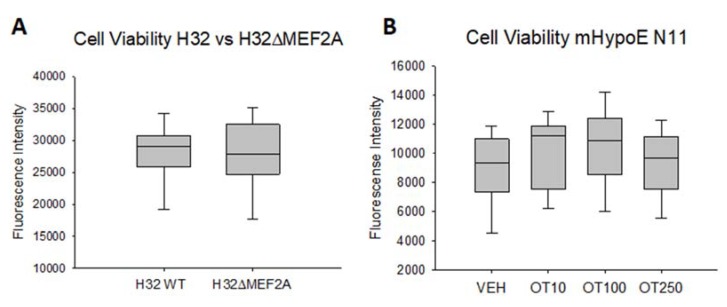
(**A**) Cell viability in H32 vs. H32ΔMEF2A. n = 24 per group. (**B**) Cell viability in OT-treated mHypoE-N11 cells. *n* = 22–24 per group.

Furthermore, we provide evidence that a knockout of MEF2A has fundamental effects on mitochondrial functioning. While the basal respiration remains unaffected, the induced maximal respiration and spare respiratory capacity is significantly disinhibited compared to the wild-type cells. Next, we asked whether the increased respiratory capacity correlates with the ATP levels available for the cells. Interestingly, we found a significantly higher cellular ATP content under basal conditions in the H32ΔMEF2A cells. Although basal respiration does not differ between the two cell types, the increased respiratory capacity of the H32ΔMEF2A cells allows them to produce more ATP on demand, mainly due to the use of the more efficient aerobic pathway (see Energy Map Figure A1). Whether the activity of MEF2A influences mitochondrial morphology or motility, and thereby respiratory functionality, remains to be addressed by future studies. Whatever the effects on mitochondrial morphology and motility might be, the elevated ATP level present in the H32ΔMEF2A cells is pivotal for the OT-induced neurite outgrowth. Changes in neurite outgrowth and plasticity are strongly intertwined processes implicated in the regulation of neuronal connectivity. Recent findings indicate that a state of neuronal hyperconnectivity may play an essential role in the etiology of ASD [19,20,62]. Many genes which have been associated with ASD and changes in neuronal connectivity belong to the integrin family [63] and Ingenuity Pathway Analysis in myotube cells with MEF2 downregulations revealed that integrin signaling is one of the few pathways targeted by all MEF2 isoforms [64]. One member of this family is integrin β1, a cell surface receptor linking the actin cytoskeleton with the extracellular matrix (ECM), mediating cell adhesion and thereby information that influences cellular morphology [65]. We showed that this receptor is significantly downregulated in the H32ΔMEF2A cells. The effects of the MEF2A knockout on mitochondrial function and integrin signaling might not be causally connected. However, it has been suggested that alterations in mitochondrial function and integrin-mediated cell adhesion are functionally coupled processes [65]. Crosstalk between cell adhesion and mitochondria is feasible, since it has been shown that mitochondria react to ECM composition changes, and in turn, modifications of the ECM can result as a consequence of mitochondrial functioning [66]. Mitochondria play a role in the mechanosensing processes of cells since mechanical forces from the ECM are transmitted to mitochondria via focal adhesions [67]. Therefore, it might be possible that the MEF2A knockout directly affects mitochondria by targeting the mitochondrial transcriptome or indirectly via mechanobiological changes transmitted by integrin receptors into the cells.

Taken together, our results reveal a central role for MEF2A in the OT-induced regulation of neuronal morphology, connectivity, and mitochondrial function.

## 4. Materials and Methods

### 4.1. Cell Culture

Rat hypothalamic H32 cells ([48] kindly provided by Prof. Greti Aguilera (NICHD, Bethesda, MD)) and mouse hypothalamic mHypoE-N11 cells (kindly provided by Prof. Eugen Kerkhoff, University of Regensburg) were cultured in DMEM (#D8437, Sigma Aldrich, Darmstadt, Germany), supplemented with 10% heat-inactivated Gold fetal bovine serum (#FBS-HI-11A, Capricorn, Germany), and Penicillin/Streptomycin (#P4333, Sigma Aldrich, Darmstadt, Germany) at 37 °C and 5% CO_2_ until 80% confluency. Passaging was performed at least once a week by gentle trypsinization. Cells were counted and seeded at 3 × 10^6^ cells/75 cm^2^ density. Cell counts were recorded and compared between wild-type and knockout cell lines to extrapolate proliferation rates.

### 4.2. CRISPR-Cas9 Mediated Knockout of MEF2A

The rat hypothalamic neuronal cell line H32 served as host to create an MEF2A knockout cell line (H32ΔMEF2A), using the Alt-R^®^ CRISPR-Cas9 system (IDT, Coralville, USA). RNP complexes were composed of the functional gRNA duplex, containing the sequence-specific crRNA (sequence ID: Rn.Cas9.MEF2A.1.AC; 5′-A*C*A*G*A*C*C*T*C*A*C*G*G*T*A*C*C*A*A*A-3′) and the ATTO 550-labeled tracrRNA in nuclease-free duplex buffer, and the S. p. Cas9 Nuclease 3NLS. The complexes were transfected by cationic lipid delivery using Lipofectamine^™^ RNAiMAX Transfection Reagent (Invitrogen, Carlsbad, CA, USA) and Opti-MEM (Gibco, Waltham, MA). Transfected single-ells were obtained 2 h after transfection by cell sorting and recultured for several weeks. The genotype was assessed by means of Western blotting.

### 4.3. Transfection of H32 and mHypoE-N11 Cells with MEF2A Overexpression Plasmids

For morphological analyses, cells were transfected with plasmids (VectorBuilder, Neu-Isenburg, Germany) by electroporation using the manufacturer’s nucleofector (Thermo Scientific, Waltham, MA) protocol. In total, 1 million cells were transfected with 0.5 µg of rat/mouse MEF2A plasmids. After a 48 h incubation, cells were further processed. The mouse hypothalamic cell line mHypoE-N11 was transfected with wild-type mouse MEF2A, resulting in mHypoE-N11^MEF2A^ cells. H32ΔMEF2A cells were transfected with either the wild-type variant of MEF2A (H32ΔMEF2A^MEF2A^) or a phospho-mimetic MEF2A mutant (H32ΔMEF2A^MEF2A[S408D]^).

### 4.4. Cell Stimulations

Cells were stimulated overnight with 10, 100 or 250 nM of OT (Bachem, Bubendorf, Switzerland) in cell culture dishes or chamber slides (BD Falcon, Germany). For the inhibition of CaN, CaN autoinhibitory peptide (#1891, Tocris, Wiesbaden, Germany) was used at a concentration of 10 µM [68]. Cells were pretreated with the inhibitor for 30 min and stimulated with the corresponding treatment. Non-treated cells are indicated as vehicle (VEH).

### 4.5. Protein Isolation

For the extraction of proteins from OT- or vehicle (H2O)-stimulated 80% confluent H32 cells, the stimulation medium was removed, and cells were washed with 5 mL ice-cold PBS (#D8537, Sigma Aldrich). Cells were scraped in 1 mL ice-cold PBS using a cell scraper (#83.1830, Sarstedt, Nümbrecht, Germany), collected in 15 mL tubes in PBS, centrifuged at 300 g and 4 °C for 5 min, and then the resulting cell pellet was resuspended in 30–100 μL RIPA lysis buffer (R0278, Sigma Aldrich, Darmstadt, Germany) supplemented with HALT inhibitor and EDTA (78444, Thermo Fisher, Waltham, MA) to obtain whole cell lysates.

### 4.6. Western Blotting

Western blotting was conducted as previously described in Meyer at al. 2018. Briefly, 15 or 20 μg of whole cell extract were loaded and separated in a 12% Mini PROTEAN or Criterion TGX Stainfree gel (Bio-Rad, Feldkirchen, Germany), respectively. Semi-dry blotting of separated proteins to nitrocellulose membranes was conducted using the FAST Blotter by Bio-Rad. Uniform total protein loading was controlled for by the Stainfree total protein method by Bio-Rad. Specific antibodies for MEF2A, pMEFA S408, pMEK 1/2 and integrin ß1 (Table 2) indicated activity and expression levels of the protein of interest.

### 4.7. Immunofluorescence

mHypoE-N11 cells and mHypoE-N11^MEF2A^, as well as H32ΔMEF2A and H32ΔMEF2A^MEF2A^ cell lines, were seeded on chamber slides (BD-Falcon, #08-774-25) at 6 × 10^4^ cells per chamber and cultured overnight in growth medium at 37 °C/5% CO_2_. Next, the growth medium was removed, and cells were briefly rinsed with warm PBS. The PBS was removed and replaced with 500 µL ice-cold 3% glyoxal solution (+20% EtOH, Acetic acid, pH = 4–5) for 20 min. After washing the cells twice with PBS-T (0,5% tween), unspecific binding sites were blocked (PBS+0,5% TritonX, 1% glycine, 10% BSA, 1% normal goat serum) for 30 min, and antibody incubated overnight at the corresponding dilution. After intense washing with PBS-T, secondary antibody (CF488A highly cross-adsorbed #20123 BioTrend, Köln, Germany) was incubated at 1:500 in PBS for 30 min. After washing, cells were incubated with Phalloidin-iFluor 594 Reagent (abcam ab176757), mounted with ProLong Glass-Antifade mounting medium with DAPI (Thermo Scientific, #P36982) and covered by cover slips (Thor Labs, refraction index 1,5; #CG15KH). Images were taken on a Zeiss AiryDisc Confocal microscope with a 63× objective with identical laser and gain intensities to guarantee comparability between images.

### 4.8. Morphological Characterization

For nucleus staining, Hoechst 33342 stain (H1399, Thermo Scientific, Waltham, MA) was added to the stimulation medium in the 6-well plates where cells were stimulated, and incubated for 30 min at 37 °C. The cells grown in cell culture dishes were washed with 1 mL PBS and subsequently fixed by gently adding 3% glyoxal solution for 20 min (3% glyoxal in H2O, #128465, (Sigma Aldrich, Darmstadt, Germany), supplemented with 20% EtOH and acetic acid to acquire pH = 5). All pictures were taken using the ZOE Fluorescence microscope (Bio-Rad, Feldkirchen, Germany). Neurite outgrowth was determined by manually tracing the length of the longest neurite per cell (using ImageJ Fiji version 1,52r software, NIH, USA) for all cells in a field that had an identifiable neurite and for which the entire neurite arbor could be visualized. Length of the neurite was measured from the edge of nucleus to the apical end of the projection. Only neurites that did not contact other cells were evaluated. At least two members of the team independently evaluated neurite length in a blinded manner.

### 4.9. Cell Viability Assay

Cellular viability was tested using the PrestoBlue Cell Viability Assay (A13261, Invitrogen, Carlsbad, CA) according to the manufacturer’s protocol and as described in [24]. Briefly, 10 × 10^3^ cells per well were seeded the day before the test in a 96-well plate in growth medium. The volume of the treatment and medium for the stimulation was calculated to a total of 90 μL per well. In total, 10 μL of PrestoBlue Reagent was added directly to the cells and incubated for 30 min before reading the fluorescence intensity with a FluoStar Plate reader (BMG Labtech, Ortenberg, Germany).

### 4.10. Mitochondrial Respiration Analysis

The oxygen consumption rate (OCR) of intact H32 and H32ΔMEF2A cells was measured with the Agilent Seahorse XF Cell Mito Stress Test Kit (Agilent Technologies, Waldbronn, Germany) according to the manufacturer’s protocol. The live cell assay monitors OCR in real time and assesses the key parameters of mitochondrial function. Mitochondrial stress compounds used for this assay are oligomycin (1 µM), carbonyl cyanide-4 (trifluoromethoxy) phenylhydrazone (FCCP; 2 µM), and rotenone/antimycin A (1 µM). On the day before the measurement, 20 × 10^3^ cells were seeded in matrigel-coated XFp 8-well miniplates (Agilent Technologies, Waldbronn, Germany) at 37 °C, humidified air and 5% CO_2_. Cartridges were prepared according to the provided protocol. The obtained values were normalized to the cell number present in the region of interest, reflecting the field of measurement by ImageJ. Data was analyzed using the WAVE software (Agilent Technologies, CA, USA) according to the manufacturer’s protocol.

### 4.11. CellTiter-Glo 2.0 Assay

The CellTiter-Glo 2.0 assay (Promega) was conducted to measure cellular ATP content under basal/non-stimulated conditions in H32 and H32ΔMEF2A cells, according to the manufacturer’s protocol. Briefly, 10 × 10^3^ cells per well were seeded the day before in a Nunclon Delta Surface 96-well plate (Thermo Scientific, #136101). On the next day, an ATP standard curve was generated, and an equal volume of Cell Titer Reagent 2.0 was added to the culture medium present in each well. After a 12 min incubation, luminescence intensity was recorded with the GloMax Luminometer (Promega, Waldorf, Germany). The assay was repeated three times.

### 4.12. Statistical Analysis

Statistical analyses were performed using Sigma Plot (version 13.0, Systat Software, Erkrath, Germany). Parametric data was analyzed by t-test or one-way analysis of variance (ANOVA) followed by the Holm–Sidak post hoc test. Non-parametric data was analyzed by the Mann–Whitney Rank Sum test or Kruskal–Wallis ANOVA on ranks and the Tukey post hoc test. Statistical significance was accepted at p < 0.05. In morphological experiments, n represents the number of cells. In Western blotting, n represents the number of cell lysates. In the ATP and cell viability assays, n is number of wells. Parametric data is presented as the mean + standard deviation (SD). Non-parametric data is represented in box plots as the minimum, first quartile, median, third quartile, and maximum. Details on statistics have been summarized in the Supplementary Materials.

## Figures and Tables

**Table 1:** Summary of cell lines used and created in this study. MEF2A protein levels have been assessed by means of Western blotting (Figure A3). mHypoE-N11 are derived from mouse hypothalamus neurons and lack endogenous MEF2A expression. Plasmid transfection caused MEF2A overexpression in those cells (mHypoE-N11^MEF2A^). H32 cells are immortalized neurons derived from a rat hypothalamus [48] and show moderate endogenous expression of MEF2A (wild-type). CRISPR-Cas directed knock out of MEF2A produced H32ΔMEF2A cells, retransfection of intact MEF2A into H32ΔMEF2A cells rescued MEF2A expression (H32ΔMEF2A^MEF2A^).

Cell Line	mHypoE-N11 (Mouse)		H32 (Rat)			
Manipulation	Wild-type	mHypoE-N11^MEF2A^	Wild-type	H32ΔMEF2A	H32ΔMEF2A^MEF2A^	H32ΔMEF2A^MEF2A[S408D]^ (inactive)
Level of MEF2A	**−**	**++**	**+**	**−**	**++**	**++**

**Table 2:** Summary of antibodies used in this study with corresponding dilution and host.

Antibody	Company and CAT Number	Dilution and Diluent	Secondary Antibody
MEF2A total	Acris AP06372PU-N	1:2000 in 5% MP	HRP-coupled anti-rabbit
pMEF2A S408	CusAb PA000728	1:5000 in 5% BSA	HRP-coupled anti-rabbit
pMEK1/2 4199	Cell Signaling 9154	1:5000 in 5% BSA	HRP-coupled anti-rabbit
Integrin ß1 (N-20)	Santa Cruz sc-6622	1:1000 in 5% BSA	HRP-coupled anti-goat

**Figure A3:**
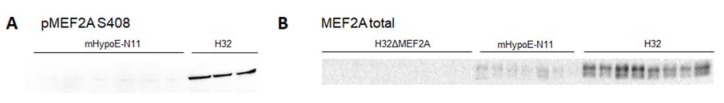
(**A**) and (**B**) Characterization of pMEF2A S408 and MEF2A total in mHypoE-N11, H32ΔMEF2A and H32 cells.

## Supplementary Materials

Supplementary materials can be found at https://www.mdpi.com/1422-0067/21/6/2200/s1.

## Abbreviations

MEF2A	Myocyte enhancer factor 2A
OT	Oxytocin
CaN	Calcineurin
MAPK	Mitogen-activated protein kinase
